# Berberine Alleviates Hepatic Steatosis by Restoring CPT1α Histone Acetylation and Modulating HDAC2/SIRT2

**DOI:** 10.5812/ijpr-170783

**Published:** 2026-06-02

**Authors:** Chenye Shi, Shuai Ma, Hua Bian, Qiong Liu, Huandong Lin, Xin Gao, Haifu Wu, Hongmei Yan, Xinxia Chang

**Affiliations:** 1 Department of General Surgery, Zhongshan Hospital, Fudan University, Shanghai, China; 2 Department of Endocrinology and Metabolism, Shanghai Geriatric Medical Center, Shanghai, China; 3 Department of Endocrinology and Metabolism, Zhongshan Hospital, Fudan University, Shanghai, China; 4 Department of Anatomy, Histology and Embryology, School of Basic Medical Sciences, Shanghai Medical College, Fudan University, Shanghai, China

**Keywords:** Berberine (BBR), Carnitine Palmitoyltransferase 1α, Histone Modifications, Hepatic Steatosis

## Abstract

**Background:**

High-fat diet (HFD)-driven hepatic steatosis is associated with impaired mitochondrial fatty acid oxidation and dysregulated expression of genes that control lipid disposal. Carnitine palmitoyltransferase 1α (CPT1α), encoded by Cpt1a, is the rate-limiting enzyme for mitochondrial fatty acid β-oxidation and responds to nutritional stress. However, the chromatin mechanisms that regulate Cpt1a in diet-induced steatosis remain incompletely defined.

**Objectives:**

To map histone modifications at the hepatic Cpt1a locus and determine whether berberine (BBR) is associated with the restoration of Cpt1a transcription through histone acetylation remodeling.

**Methods:**

Male Sprague-Dawley rats were assigned to the normal diet (ND), high-fat diet (HFD), or berberine (BBR)+HFD group (n = 8 initially), with individual rats as the experimental unit. ChIP-qPCR was performed on archived liver samples (ND, n = 6; HFD, n = 8; BBR+HFD, n = 8) and normalized to input DNA. In BRL cells, TSA (100 nM) and SAHA (20 μM) were used to assess HDAC-sensitive regulation of Cpt1a.

**Results:**

HFD reduced H3/H4 acetylation and increased H3K9 methylation at Cpt1a regulatory regions. BBR restored H3/H4 acetylation, selectively reduced H3K9me3 at +12 kb, and attenuated HFD-induced increases in HDAC2 and SIRT2 mRNA (approximately 40-fold and 3.8-fold, respectively) toward baseline. TSA and SAHA increased Cpt1a mRNA at 24 h by approximately 8.1-fold and 5.4-fold, respectively. Palmitate reduced Cpt1a expression by approximately 58%, and this repression was reversed by BBR or TSA.

**Conclusions:**

BBR alleviates HFD-induced hepatic steatosis, concomitant with the restoration of Cpt1a histone acetylation and modulation of HDAC2/SIRT2. These data support, but do not prove, an HDAC2/SIRT2-linked chromatin mechanism and should be interpreted as a candidate pathway requiring further functional validation.

## 1. Background

Metabolic dysfunction-associated steatotic liver disease (MASLD), historically termed non-alcoholic fatty liver disease, encompasses a spectrum ranging from simple steatosis to steatohepatitis, cirrhosis, and hepatocellular carcinoma (1). Current estimates indicate that MASLD affects approximately 30% of the global population and is particularly prevalent among individuals with obesity (1). Disease progression is driven not only by triglyceride accumulation but also by lipotoxicity, endoplasmic reticulum stress, mitochondrial dysfunction, and altered hepatic energy handling (2-4). Therefore, strategies that improve fatty acid oxidation (FAO) and hepatic lipid disposal remain biologically relevant therapeutic approaches.

CPT1α is a key FAO enzyme that regulates the mitochondrial entry of long-chain fatty acids, and Cpt1a responds to nutritional cues through epigenetic mechanisms. HFD can alter Cpt1a transcription through DNA methylation, histone methylation, and transcription factor recruitment (5, 6), whereas histone acetylation generally promotes transcriptional activation of metabolic genes (7). Because CPT1α activity directly influences hepatic lipid flux, chromatin changes at the Cpt1a locus may be important for understanding diet-induced steatosis. In particular, reduced histone acetylation may limit the accessibility of transcriptional regulators to metabolically important loci, whereas restoration of acetylation may help recover gene expression. However, the contribution of histone acetylation remodeling to Cpt1a repression in this setting remains incompletely defined.

BBR has anti-steatotic activity in experimental and clinical studies (8-11). In our previous rat model, BBR improved HFD-induced hepatic steatosis and increased hepatic Cpt1a/CPT1α expression; however, no detectable change in DNA methylation was observed at the Cpt1a promoter (10). This finding suggests that other epigenetic layers, particularly histone modifications, may contribute to the BBR-associated recovery of Cpt1a transcription. Clarifying this mechanism is relevant because BBR has been linked to multiple metabolic pathways, and distinguishing DNA methylation-dependent from histone-dependent effects may help explain gene-specific responses.

## 2. Objectives

We aimed to map histone acetylation and H3K9 methylation at the Cpt1a locus in HFD-fed rats, assess whether BBR restores these marks, and test Cpt1a responsiveness to HDAC inhibition in vitro.

## 3. Methods

### 3.1. Animal Experiments

Animal procedures followed our previous protocol (10). Male Sprague-Dawley rats were allocated before acclimatization by an animal caretaker using a computer-generated sequence to ND (n = 8) or HFD (n = 16; 20% carbohydrate, 60% fat, and 20% protein by calories). After 8 weeks, HFD rats were re-randomized to receive BBR chloride treatment (Sigma-Aldrich, Cat. No. B3251; purity 97 - 102% by titration and ≥ 98% by TLC; 200 mg/kg/day by oral gavage) or vehicle (0.5% methylcellulose) for an additional 16 weeks. ND rats received vehicle throughout the study.

The experimental unit was the individual rat. Group identity was coded during treatment administration, sample processing, outcome assessment (including ChIP and qPCR), and statistical analysis. Rats were maintained under a 12 h light/dark cycle (lights on 7:00 - 19:00) at 22 ± 2 °C and 50 ± 10% humidity, with free access to food and water, with 3 - 4 rats per cage.

No a priori power analysis was performed; n = 8 per group was based on previous HFD rat studies. No single primary endpoint was prespecified because this was an exploratory mechanistic study focused on Cpt1a/CPT1α and histone marks. Liver triglycerides, histology, body weight, and metabolic outcomes were characterized in our earlier model but were not generated as a complete standalone dataset in the current mechanistic analysis. Therefore, the present functional interpretation relies on the established HFD/BBR model together with the new chromatin and transcriptional data. This consideration informed the tempered mechanistic interpretation of the current results.

### 3.2. Chromatin Immunoprecipitation Analysis of the Cpt1a Locus

ChIP was performed using the Beyotime ChIP Assay Kit (P2078) with a modified protocol (12). Chromatin was prepared from approximately 100 mg of frozen liver, cross-linked with 1% formaldehyde, sheared on ice to 200 - 1000 bp fragments, and diluted in ChIP Dilution Buffer with protease inhibitors. Approximately 100 μL of diluted chromatin was used per immunoprecipitation, and an aliquot from each preparation was reserved as input DNA before immunoprecipitation.

After pre-clearing with protein A/G agarose/salmon sperm DNA, chromatin was incubated overnight at 4 °C with 2 μg of antibody: anti-acetyl-H3 (Millipore, 06 - 599, RRID:AB_2115283), anti-acetyl-H4 (Millipore, 06 - 598, RRID:AB_2295074), anti-H3K9me1 (Abcam, ab176880, RRID:AB_2751009), or anti-H3K9me3 (Abcam, ab8898, RRID:AB_306848). Antibodies were selected based on available manufacturer validation for ChIP or related immunodetection applications. Immune complexes were sequentially washed with low-salt, high-salt, LiCl, and TE buffers; cross-links were reversed; and DNA was purified before qPCR. Primer sets covered the promoter, first intron, translation initiation region, and 3'-UTR.

Input DNA was processed in parallel. IgG controls were not included because archived samples were limited. ChIP-qPCR enrichment was normalized to input DNA using the 2^-ΔΔCt^ method and expressed relative to the ND group for each region; no IgG background correction was applied. Biological replicates were individual liver samples (ND, n = 6; HFD, n = 8; BBR+HFD, n = 8). This normalization approach was applied consistently for the ChIP data and corresponding figure legends. Primer sequences are listed in Table 1.

**Table 1.** Primer Sequences Used for ChIP-qPCR of the Cpt1a Locus

Region	Position/Description	Forward Primer	Reverse Primer
**Promoter**	-387 to -261 bp relative to TSS	CCAAACAGCCAAACAAACCT	AAAAGCTCTTTGCTCCATGC
**First intron**	+12 kb region	GCCGAATTAGCCAGTGAGAG	TTAAACCGCCACCTATTTGC
**Translation initiation region**	+27.2 kb region near ATG	ATCTCTCACCCCTCCTCCAG	GATCTGTTTGAGGGCTTCGT
**3′-UTR**	+61.17 kb region	CTGACTCTCGCTGCTGTGAC	TGCATTGGTAACTGCTCAGG

### 3.3. Cell Experiments

Buffalo rat liver cells were cultured in DMEM with 10% FBS at 37 °C and 5% CO_2_. For HDAC-inhibitor time-course assays, cells were treated with TSA (100 nM) or SAHA (20 μM) and collected at 0, 3, 6, 18, and 24 h for qPCR analysis. All cell experiments used three independent biological replicates (n = 3).

For the fatty acid challenge, palmitate was conjugated to fatty acid-free BSA to generate a 10 mM PA stock in 11% BSA-containing serum-free medium and then diluted to PA 100 μM. Cells were treated for 24 h with BSA control or PA with/without BBR (20 μM) or TSA (100 nM). BSA was the control condition in PA-based experiments; separate solvent-matched vehicle controls for TSA, SAHA, and BBR were not included in this design.

### 3.4. Real-Time Quantitative PCR

RNA from frozen liver or cells was extracted in Trizol, reverse-transcribed (ReverTra Ace; Toyobo), and quantified by qPCR using the primers listed in Table 2. Expression was normalized to β-actin.

**Table 2.** Primer Sequences Used for Real-time Quantitative PCR

Gene	Forward Primer	Reverse Primer
**HDAC1**	CTGGGGACCTACGGGATATT	CACTGCACTAGGCTGGAACA
**HDAC2**	TGGCCTTTCTGAGCTGATTT	CCATGGGTATGCTCCAGTCT
**HDAC3**	TTGAAGATGCTGAACCATGC	TGGCCTGCTGTAGTTCTCCT
**HDAC4**	CTCACTGCCCTTGGAACCT	ATGCTGACGCTGGAACTCT
**HDAC6**	CCACCGGCCAAGATTCTTCT	GGGTACAGCACCCTTCTTCC
**HAT1**	AGAGTGCGGTGGAGAAGAAA	CCCCAAAGAGTTGATGGGTA
**Sirt2**	CCCACACTCAACTCTCAGCA	AAACGGGACTGAAGGAAGGT
**Cpt1a**	GGACTGTGGTGCGGAGGA	GGCTCAGGCGGAGGTCAA
**β-actin**	CCTCTATGCCAACACAGT	AGCCACCAATCCACACAG

### 3.5. Statistical Analysis

Data are presented as mean ± SEM. Distributional suitability and homogeneity of variance were assessed before parametric tests. Animal-group comparisons were performed using one-way ANOVA followed by Tukey's test. Cell time-course analyses were planned as exploratory comparisons versus the corresponding control without additional multiple-testing correction. Two-tailed P < 0.05 was considered significant.

## 4. Results

### 4.1. Histone Modification Landscape at the Cpt1a Locus

**Figure 1.**
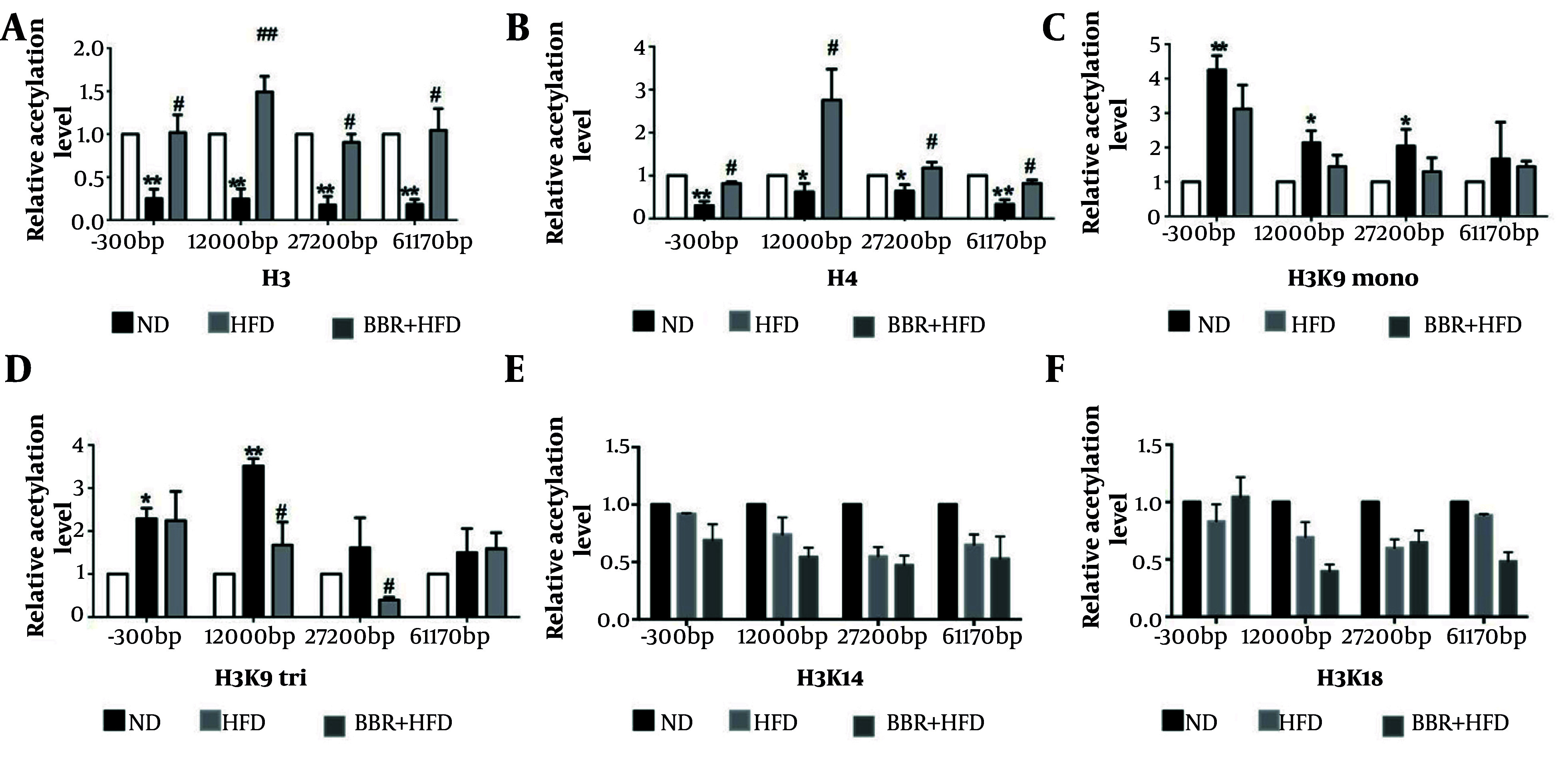
Berberine is associated with histone modification changes at the Cpt1a locus in SD rat liver. ChIP-qPCR analysis of H3/H4 acetylation, H3K9me1, H3K9me3, H3K14ac, and H3K18ac in ND, HFD, and BBR+HFD groups. Signals were normalized to input DNA by 2^-ΔΔCt^ method. Biological replicate = individual rat liver; ND, n = 6; HFD, n = 8; BBR+HFD, n = 8. Data are mean ± SEM. * P < 0.05 and ** P < 0.01 versus ND; # P < 0.05 and ## P < 0.01 versus HFD.

Because our previous work showed no change in Cpt1a promoter DNA methylation despite BBR-mediated restoration of Cpt1a/CPT1α expression (10), we profiled histone marks across a 62-kb Cpt1a region. HFD reduced H3/H4 acetylation across regulatory intervals spanning from +61.17 kb to -300 bp relative to the transcription start site, whereas BBR restored acetylation toward control levels at these sites (Figure 1A and B). These data indicate that Cpt1a repression in the HFD model is associated with reduced histone acetylation rather than promoter DNA methylation. The promoter-proximal and downstream regulatory regions showed broadly similar directional changes, supporting a coordinated chromatin response at the locus.

HFD increased H3K9me1 at -300 bp, +12 kb, and +27.2 kb and increased H3K9me3 at -300 bp and +12 kb. BBR tended to reduce H3K9me1 and selectively reduced H3K9me3 at +12 kb (Figure 1C and D). By contrast, H3K14 and H3K18 acetylation were not significantly restored by BBR (Figure 1E and F), suggesting that the chromatin response was region- and mark-specific rather than a uniform change across histone modifications.

### 4.2. Hepatic Histone Deacetylase Modulation

**Figure 2.**
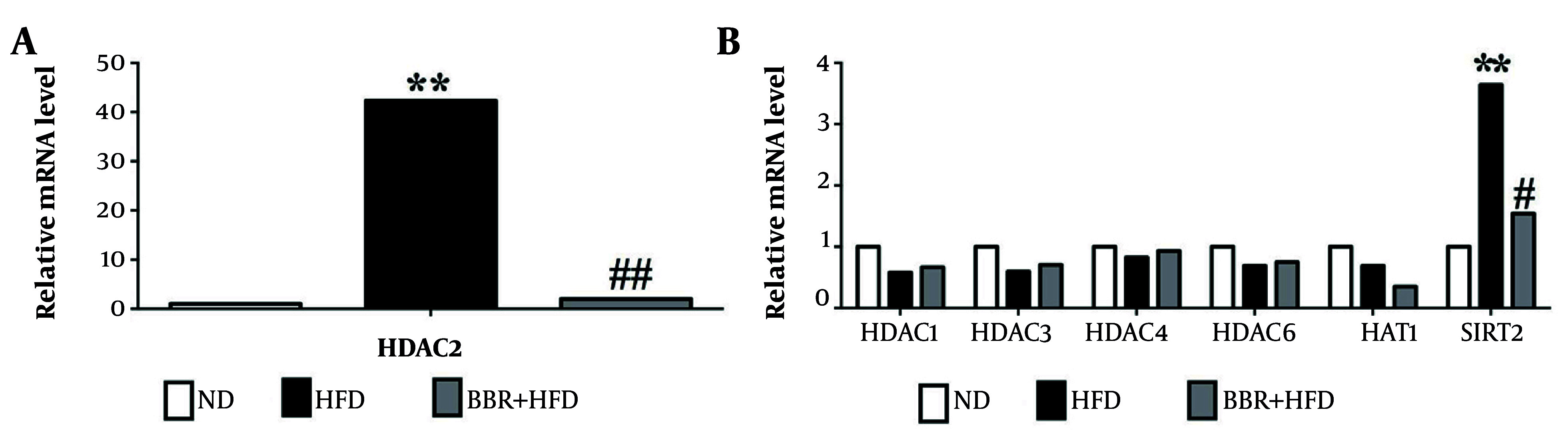
Berberine attenuates HFD-associated HDAC2 and SIRT2 upregulation in SD rat liver. qPCR values were normalized to β-actin. Biological replicate = individual rat liver; ND, n = 6; HFD, n = 8; BBR+HFD, n = 8. Data are mean ± SEM. ** P < 0.01 versus ND; # P < 0.05 and ## P < 0.01 versus HFD.

HFD increased hepatic HDAC2 and SIRT2 mRNA by approximately 40-fold and 3.8-fold, respectively, and BBR reduced both toward baseline (Figure 2A and B). HDAC1, HDAC3, HDAC4, HDAC6, and HAT1 were not significantly changed. These results suggest that the HFD/BBR response was not a generalized change across all measured histone-modifying enzymes but was most evident for HDAC2 and SIRT2.

### 4.3. Cpt1a Transcriptional Activation in Vitro

**Figure 3.**
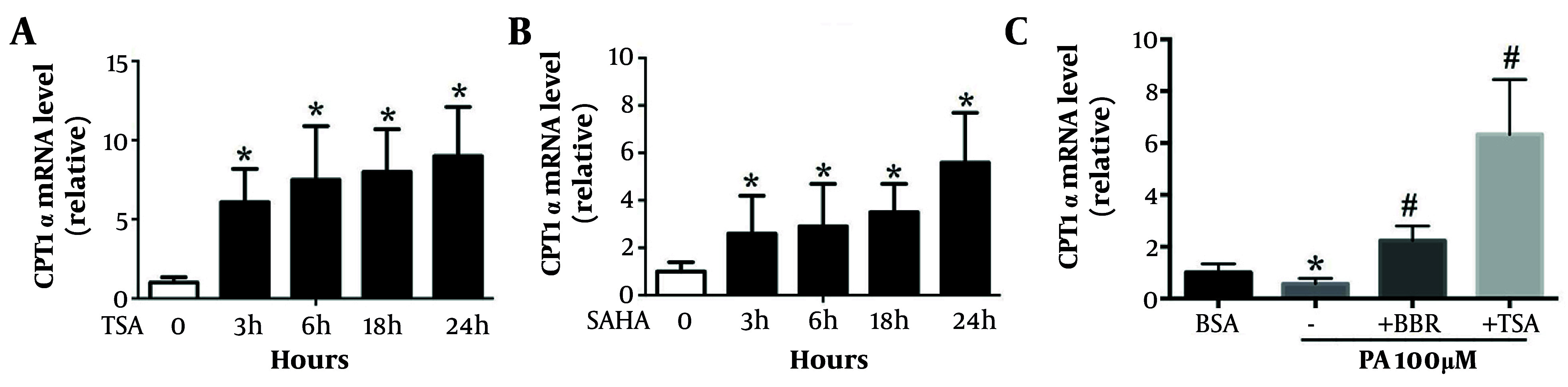
Berberine and HDAC inhibitors promote Cpt1a/CPT1α transcription in BRL cells. Cells were treated with TSA (100 nM) or SAHA (20 μM) for 3 - 24 h (A,B), or with BSA control or PA (100 μM) plus BBR (20 μM) or TSA (100 nM) for 24 h (C). Independent experiments, n = 3. Data are mean ± SEM. A,B: *P < 0.05 versus 0 h. C: * P < 0.05 versus BSA; # P < 0.05 versus PA alone.

TSA (100 nM) and SAHA (20 μM) increased Cpt1a/CPT1α mRNA in a time-dependent manner, peaking at 24 h (approximately 8.1-fold and 5.4-fold; Figure 3A and B). PA reduced Cpt1a expression by approximately 58%; this repression was reversed by BBR or TSA (Figure 3C). These results support HDAC-sensitive regulation of Cpt1a transcription in hepatocytes and provide cellular support for the animal ChIP findings. However, these data alone do not establish a direct causal requirement for HDAC2 or SIRT2.

## 5. Discussion

This study demonstrates that BBR treatment is associated with the restoration of histone acetylation at the hepatic Cpt1a locus and modulation of HDAC2/SIRT2 expression in HFD-fed rats. Together with the in vitro HDAC-inhibitor data, these findings support an HDAC-sensitive chromatin component in Cpt1a regulation; however, they remain associative and do not establish a direct requirement for HDAC2 or SIRT2. This distinction is important because pharmacological HDAC inhibition can affect multiple enzymes and pathways, and the current data do not identify which deacetylase is necessary for the observed transcriptional response.

CPT1α is central to hepatic FAO and lipid homeostasis (5, 13). Increasing hepatic CPT1α activity protects against lipid accumulation in experimental models (14, 15), whereas HFD or fructose feeding can suppress Cpt1a through epigenetic changes (6, 16). Our data extend these observations by showing reduced H3/H4 acetylation and increased repressive H3K9 methylation at selected Cpt1a regulatory regions in HFD-fed rats, with partial reversal after BBR treatment. The most pronounced acetylation changes were observed across multiple Cpt1a regions, supporting a locus-wide response rather than an isolated promoter-only event.

Cpt1a regulation appears context dependent. Prior work linked Cpt1a methylation to metabolic traits and fructose-induced hepatic dysregulation (16-18), whereas our rat model showed no change in promoter DNA methylation at Cpt1a (10). The present ChIP data therefore support histone acetylation as an additional regulatory layer. Increased H3K9me1/me3 may also contribute to transcriptional repression, although BBR did not broadly reverse all methylation marks. This pattern suggests that BBR acts primarily by restoring acetylation, with a more selective effect on repressive methylation. Such mark-specific regulation is consistent with the concept that distinct epigenetic modifications can respond differently to diet and pharmacological intervention.

BBR has multiple metabolic actions (8-11, 21). At the Mttp locus, BBR affected DNA methylation (10); at Cpt1a, it primarily restored histone acetylation and normalized HDAC2/SIRT2 expression. HDAC2 has been discussed as a target for liver disease (22), and SIRT2-linked signaling has been implicated in experimental MASLD (23). The parallel changes observed here identify HDAC2 and SIRT2 as candidate associated deacetylases; however, direct loss-of-function studies, selective enzyme inhibition, or ChIP assays assessing HDAC2/SIRT2 occupancy at Cpt1a are needed to establish causality. Future studies combining enzyme-specific perturbation with FAO flux measurements would help link chromatin changes to metabolic function.

Limitations include the use of a single HFD rat model, incomplete new phenotypic endpoints in this mechanistic cohort, the absence of IgG ChIP controls and solvent-matched vehicle controls, the lack of FAO flux measurements, and no direct HDAC2/SIRT2 functional testing or human tissue validation. These limitations are important because they constrain the strength of mechanistic inference and support interpreting HDAC2/SIRT2 as candidate mediators rather than established causal drivers. Nevertheless, the consistency between the animal ChIP data and cell-based HDAC inhibitor responses supports the overall trend of acetylation-sensitive Cpt1a regulation. Additional studies using enzyme-specific knockdown or inhibition, input-normalized ChIP with IgG controls, and direct FAO flux assays would help determine whether these chromatin changes are necessary for the metabolic effects of BBR.

### 5.1. Conclusions

In summary, HFD-associated Cpt1a/CPT1α repression coincided with reduced histone acetylation and increased H3K9 methylation at selected regulatory regions. BBR restored acetylation, selectively attenuated H3K9me3 at +12 kb, and modulated HDAC2/SIRT2 expression. These findings support an HDAC2/SIRT2-linked chromatin pathway as a candidate mechanism for BBR-associated regulation of hepatic FAO genes, while emphasizing the need for targeted validation in future studies.

## Data Availability

The datasets generated and/or analyzed in the present study are available from the corresponding author upon reasonable request.
